# Abstracts and Items

**Published:** 1859-01

**Authors:** 


### ABSTRACTS AND ITEMS.

PREPARED BY THE JUNIOR EDITOR.

### BURNS.

Two parts of collodion and one of castor oil make an excellent mixture for burns. The mixture should be spread on with a camel's hair pencil. It forms a cuticle that protects the parts from the air and other irritants, and is perfectly painless. It may be allowed to remain until suppuration begins, when a poultice of light bread and water will remove it. The healthy granulating surface may be dressed with simple means, and readily heals. Should cracks occur in this artificial cuticle, a little more of the article will close them up. Two coats put on the first and second day will answer the purpose.

### NEURALGIA.

Prof. Crawcona, of New Orleans, in the *Hospital Gazette*, recommends subcutaneous injection of solution of morphia as a remedy for obstinate neuralgia. Quarter grain of sul. morph. dissolved in ten drops of water, by means of a fine syringe injected under the skin, in the neighborhood of the part affected, repeated once or twice in twenty-four hours.

### PUERPERAL CONVULSIONS.

Venesections, cathartics with castor oil, cold to the head, chloroform and delivery, are the tested and attested remedies for this dreadful complication of labor.

### SALINE TREATMENT FOR DYSENTERY.

First give a cathartic dose of sul. magnesia, and follow with a solution of one ounce of the sulphate to six ounces of water, in teaspoonful doses, every two hours. It will be necessary to lengthen out the time of giving the solution to three, four or six hours, or twice a day, as the cure proceeds, to prevent too frequent movement of the bowels. This simple treatment in uncomplicated cases, begun early, is often sufficient to effect a complete cure.

### SORE NIPPLES.

Two parts of collodion and one of castor oil make an excellent artificial cuticle for cracked nipples. One part of prepared chalk to two parts of rosin and simple cerate make an excellent ointment for them. One drachm of tannin to an ounce of glycerine will often have a good effect upon them. In chronic cases, a single application of a wash of sixty grains of crystalized nitrate of silver to half an ounce of pure water will often dispose them to heal. The application should not be repeated in less than four days, if it should become necessary to repeat at all.

### APOCYNUM CANNABINUM FOR AGUE.

Dr. Trent, in the *Southern Medical and Surgical Journal*, recommends the following formula as an excellent remedy for chills:

| | | |
|---|---|---|
| ℞ | Apocynum cannabinum, | ʒj. |
| | Olei mg. pip., | guttæ xvi. |
| | Syrup, | q. s. |

Mix, ft. pil. No. xii. One to be taken every two hours in time to take four before the expected paroxysm, and thus repeated until they cease to return. The twelve are generally enough. It is well to precede them with an anti-bilious cathartic, but is not always necessary. He details several cases cured in this way.

### TETANUS (TRAUMATIC).

Dr. G. A. Jones, of Texas, reports a case in the *Nashville Journal* of this formidable disease cured by a mixture of chloroform and sul. ether; at least, we think they were the essential means used. He administered the mixture by inhalation, so as to keep his patient relapsed below the point of spasmodic action for eight days. The convulsions gradually diminished after the third day until on the eighth they entirely disappeared. His other treatment consisted of sinapisms to spine, calomel and compound extract of colocynth as cathartic second day, injections, castor oil to keep bowels soluble. Towards the close, nourishment and tonics were used. The patient was anestheticated at the approach of every paroxysm of convulsions without either regard to quantity or frequency of application of the means.

### ASCARIDES.

Dr. Schultz recommends a clyster formed of argenti nitras grs. x. to xv., aqua distil. ℥iv., to be used for ascarides. It may be repeated every day until it succeeds.

### INFANTILE CONVULSIONS.

Prof. Simpson uses chloroform very freely in infantile convulsions.

### ALBUMINURIA.

Dr. C. Lees, in the Dublin *Hospital Gazette*, recommends a persevering treatment of albuminuria, with iron and occasional cathartic of jalap, a vapor bath once a week and flannel next the skin. He records a case cured. The treatment should be continued for several months.

### PEDICULUS PUBIS.

Use a lotion of bi-chloride of mercury twelve grains, alcohol and distilled water each two ounces (or whisky four ounces).—Dr. Ryding, *Lancet*.

### ASH TEA AS A REMEDY FOR THE BITE OF A RATTLESNAKE.

A correspondent of the Nashville *Journal of Medicine and Surgery* affirms that a tea prepared from the bark of ash is a reliable remedy for the bite of the rattlesnake. He does not give the variety of the ash tree used, nor very definite directions as to its preparation. His treatment, however, is the administration of "about one pint of ash tea, prepared by taking a handful of the inner bark of the ash, adding one quart of water, and boiling down to a pint." About half a gill is to be taken every twenty minutes.

### RIGID OS UTERI.

Tartar emetic in one-eighth of a grain doses every hour will often relax an obstinately rigid os uteri in a much shorter time than it would naturally yield. Look out for all obliquities in cases where the os is a long time dilating, particularly in multipara.

### COLLODION IN HERPES ZONA.

Prof. Fenger has of late been treating this troublesome affection advantageously by collodion, smearing it, by means of a pencil, over the whole of the vesicles, their bases and circumference, or wherever there is redness. It should be applied as early as possible, and three layers in thickness, renewing it next day. He finds the addition of castor oil to the collodion an improvement; but especially prefers the solution of cotton wool in acetic ether.—*Schmidt's Jahrb.: Med. Times and Gazette.*

### ERUPTION ON EDGE OF EYELID.

℞ Hyd. precpt. rub.,
Ung. simp., aa. pt. 1.

Pulverize thoroughly the first article and mix intimately with the latter. Apply at bedtime to the affected parts, being careful not to touch the conjunctiva; or touch each pustule with strong tincture of iodine. When this last is used, the lids should be everted so as not to touch the eye. Or with a pointed stick of nitras argenti touch each pustule separately; or touch the edges of the lid all along the affected part with sul. cupri. Before making any of these applications, the scabs and scales of cuticle should be carefully removed by tepid water, a fine probe or tooth pick.

